# The Migraine-Anxiety Comorbidity Among Migraineurs: A Systematic Review

**DOI:** 10.3389/fneur.2020.613372

**Published:** 2021-01-18

**Authors:** Leila Karimi, Tissa Wijeratne, Sheila Gillard Crewther, Andrew E. Evans, Deena Ebaid, Hanan Khalil

**Affiliations:** ^1^School of Psychology and Public Health, College of Science, Health and Engineering, La Trobe University, Melbourne, VIC, Australia; ^2^Department of Neurology, Western Health & University Melbourne, AIMSS, Level Three, WHCRE, Sunshine Hospital, Melbourne, VIC, Australia; ^3^Department of Medicine, Faculty of Medicine and Allied Sciences, Rajarata University of Sri Lanka, Anuradhapura, Sri Lanka; ^4^Royal Melbourne Hospital, Melbourne, VIC, Australia; ^5^The Florey Institute of Neuroscience and Mental Health, Parkville, VIC, Australia

**Keywords:** migraine, anxiety, systematic (Literature) review, prevalence, comorbidity

## Abstract

**Background:** Migraine is recognized as a neurological condition that is often associated with comorbid psychiatric symptoms such as anxiety, depression, bipolar disorder and/or panic disorder. Though some studies have demonstrated the link between migraine and anxiety disorders, there are no systematic reviews that have been published in this area to summarize the evidence. The aim of the present study is to systematically review the literature associated with comorbidity of migraine and anxiety disorders among migraineurs compared to non-migraineurs.

**Methods:** The present systematic review included population-based, cohort and cross-sectional studies if they were reporting the frequency of migraine with either anxiety or depression as diagnosed by a medical practitioner according to the International Classification of Headache Disorders (ICHD-2/3).

**Results:** Eight eligible studies from 2060 relevant citations were included in the review. All participants were migraine patients from both primary care and outpatient settings, as well as tertiary headache and anxiety centers, and were compared to non-migraineurs. The results of the systematic review showed that there is a strong and consistent relationship between migraine and anxiety. The co-morbidity of co-occurrence for migraine and anxiety has an average OR of 2.33 (2.20–2.47) among the prevalence and cross sectional studies and an average RR of 1.63 (1.37–1.93) for two cohort studies; The major limitations of included studies were small sample sizes and a lack of adjusting of confounding factors.

**Conclusion:** The results highlight the need for inclusion of an anxiety screening tool during initial assessments of migraine patients by medical practitioners and/or physicians and may explain why some anxiolytic medications work better than others for migraine mitigation.

## Introduction

Migraine and other headache disorders are among the most prevalent disorders worldwide (1). Migraine is a debilitating headache disorder that is usually diagnosed by medical practitioners based on clinical history and the exclusion of other headache types. There are broadly two main types of migraine; one with aura, and one without (2). The US statistics on the migraine prevalence rate show that one in every seven Americans suffer from migraine or severe headache annually (3). The findings of a review in Europe demonstrated migraine prevalence to be around 14.7% with almost twice as many females (17.6%) as males (8%) (4).

Migraine is a multifactorial neurological disorder, that is associated with genetic, hormonal, environmental, dietary and psychological factors (5). Most migraine is episodic (<15 headache days per month). Chronic migraine is less common but has a high personal, familial, and social impact. The diagnosis is made when there are at least 15 headache days monthly including 8 migraine days per month for at least 3 months. The prevalence is 1.4–2.2% in the population (6). Given the frequency of intensely painful headaches, it is not surprising that chronic migraine is often associated with common psychiatric disorders such as anxiety disorders (7). Generalized anxiety disorder is **c**haracterized by emotionally unpleasant developmentally inappropriate states of unfocused uneasiness and worry, usually about objectively unthreatening situations (8). Generalized anxiety disorder is associated with perturbed heart rate, blood pressure, inflammation, muscular tension (9), restlessness, fatigue and problems in concentration, somatic complaints, and rumination (10).

The association of migraine and anxiety has been elucidated in both clinical as well as community-based settings (11, 12). For example, individuals with migraine showed a higher prevalence of generalized anxiety disorder even after adjusting for demographic variables and pain conditions including arthritis and back pain (10, 12). The authors also reported that chronic condition such as arthritis and pain were also more prevalent in migraineurs than those who do not have migraine (12). The authors analyzed secondary data collected from a Canadian Community Health population based survey and found that people with generalized anxiety disorder were 2.5 times higher in migraineurs than those without migraine (10). In another study conducted by Antonaci et al., the authors reported that general anxiety and social phobia were the types of anxiety which demonstrated the strongest relationship with migraine amongst young adults (11).

Since the early epidemiological study of Breslau, Davis (13), the relationship between migraine in young adults and psychiatric disorders has been understood to be bidirectional with bipolar disorder, panic disorder, or generalized anxiety disorder and alcohol and drug abuse in the majority of migraine patients (13–15). Similarly, in a study by Swartz, Pratt (16), significant associations were found between migraine and depression, panic and phobia and suicide, even after adjusting for age and sex (16). Later, Breslau et al. (14) demonstrated that the comorbidity of migraine and psychiatric disorders further increased the likelihood of disability, complicated psychiatric and neurological care (14) and significantly heightened the risk of suicide (13, 14). Indeed, a recent review of the migraine-suicide link identified migraine as risk factors for suicide attempt, even after adjusting for psychiatric conditions (17). Such findings propose a role for migraine pain as a risk factor in suicide attempts (18). In another recent systematic review (19), a bidirectional relationship between migraine, major depression and panic disorder was also highlighted.

The neurological pathophysiology of comorbid migraine and anxiety disorders has been studied clinically, often when associated with balance disorders (20, 21). Such evidence on the neurophysiology of migraine has provided insight into its association with anxiety. For example, evidence suggests that altered brainstem signaling mechanisms play a vital role in the pathophysiology of migraine, particularly in relation to symptoms such as nausea, vertigo and other autonomic symptoms (22). These symptoms are also characteristics of heightened anxiety (23, 24). A role for trigeminovascular activation has been supported by the effect of serotonin agonists such as triptans and Calcitonin Gene Related Protein antagonists in managing migraines (25). CGRP as a potent vasodilator also functions in the transmission of nociception which is inevitably tied to the stress and anxiety occasioned by frequent migraine (25).

It is important to note that acute anxiety is an innate biologically adaptive response to real potential threats in the environment. Acute anxiety is mediated by the hypothalamic-pituitary-adrenal (HPA) axis and sympathetic medullary axis (SMA) and together their interaction affects human behavior and cognition (26). By comparison, prolonged anxiety and over-activation of the HPA-axis is known to be maladaptive, leading to perturbation of the stress response (27, 28). Indeed, prolonged anxiety has been proposed as a causal factor influencing the role of neuropathologic processes and leading to increased risk of other psychiatric disorders, as well as the transformation of episodic migraines into chronic events (29, 30).

Currently, anxiety disorders are among the most common psychiatric disorders. The prevalence of anxiety disorders among migraineurs is double that associated with depression, (31, 32). It is generally acknowledged that depression and anxiety have overlapping but distinctive features that may have different neurobiological underpinnings. For instance, (33) influential tripartite model of anxiety and depression [developed by (34)] provided an extremely influential account of the similarities and differences between anxiety and depression. In terms of similarity, anxiety, and depression are both strongly associated with negative affectivity or the experience of distress and other negative emotional states. Clark and Watson also identified two other factors: (1) positive emotionality (involving energy and pleasurable engagement; it is orthogonal to negative emotionality; and (2) physiological hyperarousal. Depression (but not anxiety) is characterized by a relative absence of positive affect (or manifestation of anhedonia). In contrast, anxiety (but not depression) is characterized by hyperarousal. In contrast to the research into the relationship between depression and migraine, substantially less research is available on migraine comorbidity with anxiety (6). Furthermore, there have been few, if any systematic reviews or meta-analyses on the migraine and anxiety relationship exclusively. Thus, the aims of this review were twofold: (a) to systematically evaluate the connection between anxiety and migraine, and (b) to determine using Odds Ratios (OR) and Relative Risk Ratio (RR) whether the comorbidity of migraine with anxiety is higher in migraineurs than non-migraineurs.

## Method

The review was conducted in accordance with the Joanna Briggs Institute methodology for systematic reviews of etiology and risk (35).

### Eligibility Criteria

The inclusion criteria were studies with (1) all types of quantitative study designs, (2) participants aged 16 years and above (3) a clear diagnosis of migraine by a medical practitioner or a recorded medical history or diagnosis based on ICHDS-II/III classifications, (4) patients who experienced at least one migraine episode monthly or more severe conditions as per Weatherall (31), and (5) a comparison group of non-migraineurs.

The outcomes of interest that were considered for this review were any measures of anxiety by a clinically validated tool such as the Goldberg Anxiety Disorder (GAD), Depression, Anxiety and Stress Score (DASS-21), Goldberg Anxiety Scale (GAS), Hamilton Anxiety Scale (HAS), self-reported anxious symptomology (RAS) & Hospital Anxiety Depression Scale (HADS). Studies were excluded from the review if (1) they were not written in English language, (2) if they included participants under 16 years of age, or (3) if they did not have a non-migraineur comparison group.

### Data Sources

A systematic search was conducted on December 2019 through electronic database of Medline, PsycINFO, EMBASE (Ovid), Science Direct (Elsevier), Cochrane, and PubMed, for all available years of publication until December 2019. Reference lists of included studies were also hand searched. The following MeSH terms and Keywords were used: Migraine/or chronic or tension or intractable/or headache, Migraine disorders/or Tension type, headache/or Headache disorders and Anxiety/or Anxieties/or GAD, or Panic^*^/or Neurotic/or Neuro anxiety/or panic/or anxiety Disorders/or Panic disorders/or Neurotic Disorders.

### Study Selection

Following the search citations were entered to EndNote X9 and duplicates were removed. citations were evaluated independently by two reviewers (LK and HK). The full text of identified citations was evaluated by the two reviewers (LK and HK). Two reviewers (LK and HK) independently evaluated the studies. Reasons for exclusion of full text studies were recorded. Any reference conflicts were resolved by consensus between the two reviewers.

### Quality Assessment

The risk of bias within the citations were evaluated with the Joanna Briggs Institute (JBI) critical appraisal tools for prevalence, cross-sectional and cohort studies as shown in Tables 2–4 (35, 36). The main criteria in assessing risk of bias included the appropriateness of study design, adequacy of sample size, methods and measurements, and data analysis.

#### Data Extraction

A template for data extraction was formed using the JBI Database of Systematic Reviews and Implementation Reports. Each reviewer extracted data on half of the included studies, while the other reviewer checked the extracted the data (LK and HK). The information extracted from each individual study included; study characteristics (country, author, date of study, setting of study), participant characteristics (total number, diagnosis), outcome measures (type of anxiety tool used), and results (association between migraine and anxiety, r (p) values or Odds Ratio (95% CI) where available).

### Data Analysis

Data analysis including Odds Ratio (OR), relative risk ratio (RR) were produced using Meta-Essential (37). The meta-analysis graphs were not produced nor reported given large level of heterogenities among the studies and inadequate study size. We also undertook a sensitivity analysis by clacluating OR or RR on all the similar study types separately, (prevalence, cohort and cross-sectional studies). Based on Cochrane guideline “a sensitivity analysis is a repeat of the primary analysis or meta-analysis, substituting alternative decisions or ranges of values for decisions that were arbitrary or unclear” (38). For this study, given different study types were used in the review, in addition to calculating overall OR or RR on all combined studies, a separate calculation was conducted on each sets of study types (i.e., prevalence, cohort, and cross-sectional studies) to find out if there is any variability in the results due to different study types.

The systematic review is registered with Prospero (#CRD42020153059).

## Results

**Figure 1:**
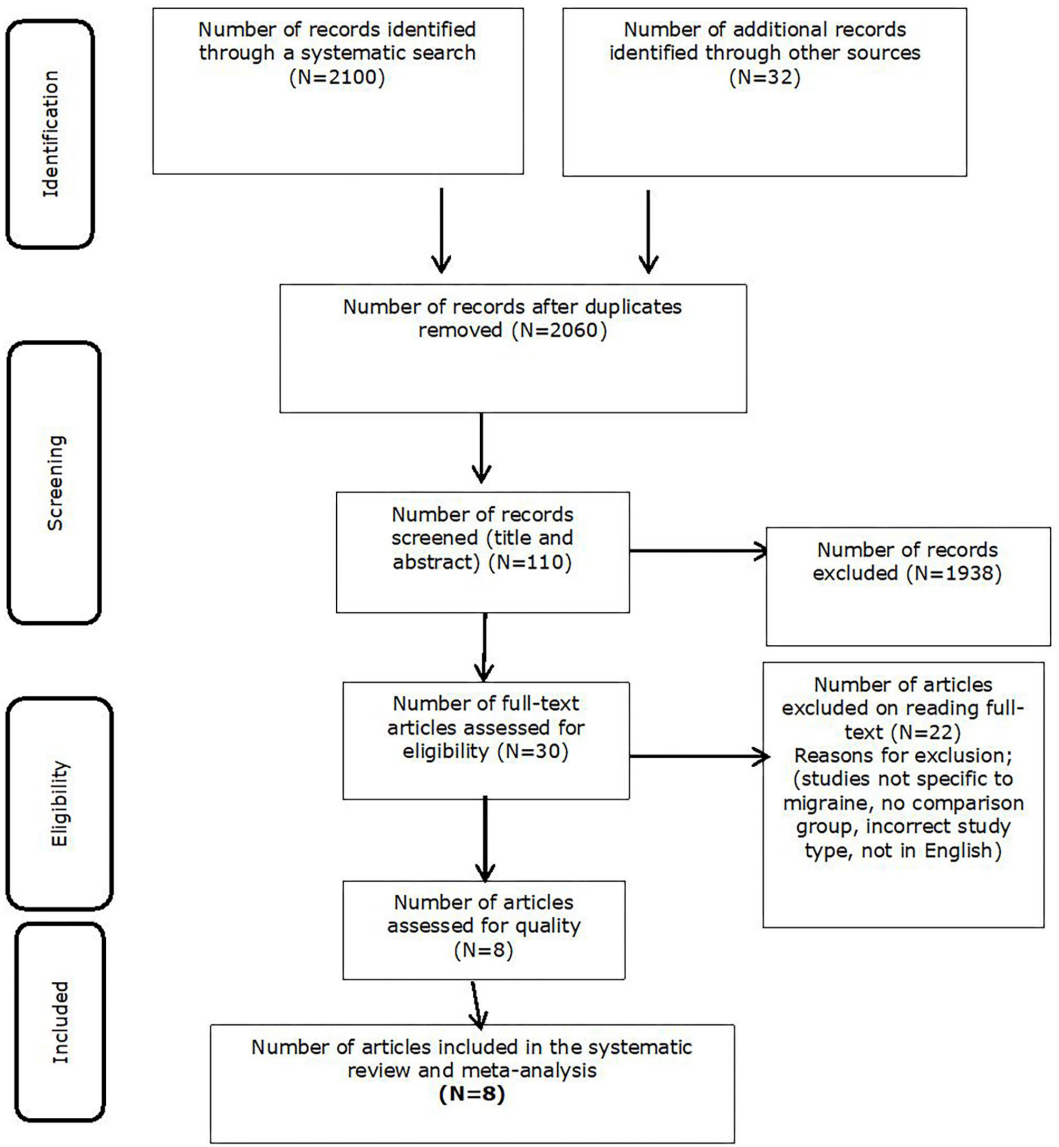
PRISMA of study selection.

The initial search generated a total of 2,132 citations. After removing the duplicates (*n* = 72), 2,060 unique citations were identified. After screening the citations (*n* = 110), 30 studies were identified as eligible for full-text review. Studies were removed if they were not written in English, participants within the study were aged under 16 years of age, incorrect study type, and/or the study did not meet risk of bias criteria. Following the full-text review, 22 more studies did not meet the eligibility criteria i.e., they did not include the minimum information required such as including a comparison group of non-migraineurs. A total of eight studies were eligible for inclusion in the review as shown in Figure 1.

### Study Characteristics

The studies were conducted in Canada, Turkey, Brazil, USA, New Zealand, Korea, China, and the European Union. The study types were population-based, cohort and cross-sectional studies. Migraine was diagnosed in the majority of the studies based on ICHDS-II/III classifications. The studies used a variety of validated tools as the screening measure for anxiety, including the Goldberg Anxiety Disorder (GAD), Depression, Anxiety and Stress Score DASS-21, Goldberg Anxiety Scale (GAS), Hamilton Anxiety Scale (HAS/HAMA), and self-reported anxious symptomology (RAS). In all the studies the results demonstrated a strong relationship in terms of Odds Ratios (38) between anxiety and migraine compared to non-migraineurs. Both the Brazilian studies (29, 39) showed exceptionally high ORs (OR = 13 and 25 in order), with the other six studies showing ORs ranging from 1.77 to 4.5 (40, 41).

The same eight studies were included in the systematic review and were characterized by both primary care and outpatient settings, as well as tertiary centers. Participants included those with ICHDS-II/III classified migraine, compared against non-migraineurs. Please see Table 1 for the study characteristics.

**Table 1:** Characteristics of included studies—prevalence, cross-sectional, and cohort studies.

**Country/References**	**Methods (data collection procedure)**	**Sample size**	**Age (years) Range/mean (SD)**	**Migraine assessment**	**Anxiety measure**	**Comorbidity of migraine with anxiety (vs. non-migraineurs) Odds ratio/Risk Ratio (95% CI)**
**Association of migraine with anxiety compared to non-migraineurs**						
Brazil/Mercante et al. (39)	The Anxiety Disorders Program of the Institute of Psychiatry	60	19–70	ICHD-II	GAD	OR 13.00 (3.45–48.93)
Turkey/Karakurum et al. (42)	n/c	87	32.3 (10.05)	IHS	Hamilton Anxiety Scale (HAS)	RR 2.10 (1.38–3.19)
European union countries/Lampl et al. (43)	Primary care-population based surveys	6,624	42.1 (12.9)	ICHD-2	HADS	OR 1.77 (1.54–2.04)
Korea/Oh et al. (44)	Primary care -population based surveys	2,762	19-69	ICHD-2	Goldberg Anxiety Scale (GAS)	OR 4.5 (3.1–6.5)
North West Pacific areas (New Zealand)/Orta et al. (40)	Primary care -pregnant women	1,321	33.1 (4.3)	ICHD_II and the deCode Genetics migraine questionnaire (DMQ3)	DASS-21	RR 1.55 (1.21–1.99) mild—sever dass
Brazil/Peres et al. (29)	Primary care self-administered questionnaire	782	34.2 (6.3)	Self-reported ICHD-II	GAD-7 (anxiety)	OR 25.16 (16.50–38.39)^†^
Canada/Senaratne et al. (41)	Outpatient anxiety clinic- computer-assisted telephone interview (CATI)	206	37.8 (12.9)	IHS	GAD	OR 1.37 (0.72–2.61)
US/Victor et al. (45)	Epidemiological national survey	30,790	43.6	Self-reported medical diagnosis of migraine	Self-reported anxious symptomology (RAS)	OR 2.30 (2.15–2.45)

† *Calculation based on corresponding with the author*.

Eight studies did not meet the minimum number of studies as well as heterogneties criteria to be included in a meta-analysis (52). Given presence of large heterogenties of the combined studies (*p* < 0.05, I^2^ > 0.75), a minimum of 40 studies are required to conduct a metanalysis (52). Therefore, only an average OR and RR for study subtypes are presented for this review. Based on the findings demonstrated in Table 1, an average OR of 2.33 (2.20–2.47) among the six studies are reported. For prevalence studies (41, 44), the OR was 2.54 (95% CI 1.48–4.35). The average OR for the cross-sectional studies (39, 46) was higher and reported to be 8.14 (95% CI 0.99–66.83). The high OR and CI for this group of studies were due to inclusion of the two Brazilian studies with very high OR. For two cohort studies an average of RR of 1.63 (1.37–1.93) reported. All the study types showed a significant association between migraine and anxiety.

### Risk of Bias in Individual Studies

#### Prevalence Studies

There was a total of three prevalence studies as shown in Table 2 (41, 43, 44). Only one study (43) fulfilled all the requirements for a high-quality study. The other two studies met the criteria for adequate sampling, valid methods for identifying the condition, and data analysis. Small sample size was the major limitation in one study (44).

**Table 2:** Risk of bias in prevalence studies.

**References**	**Was the sample frame appropriate to address the target population?**	**Were study participants sampled in an appropriate way?**	**Was the sample size adequate?**	**Were the study subjects and the setting described in detail?**	**Was the data analysis conducted with sufficient coverage of the identified sample?**	**Were valid methods used for the identification of the condition?**	**Was the condition measured in a standard, reliable way for all participants**	**Was there appropriate statistical analysis?**	**Was the responses rate adequate, and if not, was the low response rate managed appropriately?**
Oh et al. (44)	Yes	Yes	No	Yes	Yes	Yes	Yes	Yes	No
Senaratne et al. (41)	Yes	Yes	Unclear	Yes	Yes	Yes	Yes	Yes	Yes
Victor et al. (45)	Yes	Yes	Yes	Yes	Yes	Yes	Yes	Yes	Yes

#### Cross Sectional Studies

As presented at Table 3, there was a total of three cross sectional studies (29, 39, 45). All three studies had appropriate sampling, and the exposure and outcomes were assessed in a reliable way with suitable statistical analysis. None of the studies detailed any approaches to deal with confounding factors.

**Table 3:** Risk of bias in cross sectional studies.

**References**	**Were the criteria for inclusion in the sample clearly defined?**	**Were the study subjects and the setting described in detail?**	**Was the exposure measured in a valid and reliable way?**	**Were objective, standard criteria used for measurement of the condition?**	**Were confounding factors identified?**	**Were strategies to deal with confounding factors stated?**	**Were the outcomes measured in a valid and reliable way?**	**Was appropriate statistical analysis used?**
Balaban et al. (51)	Yes	Unclear	Yes	Yes	Unclear	Unclear	Yes	Yes
Mercante et al. (39)	Yes	Yes	Yes	Yes	Unclear	Unclear	Yes	Yes
Lampl et al. (43)	Yes	Yes	Yes	Yes	Yes	Unclear	Yes	Yes
Peres et al. (29)	Yes	Yes	Yes	Yes	Unclear	Unclear	Yes	Yes

#### Cohort Studies

There were only two cohort studies (40, 42) eligible for inclusion in the cohort risk of bias consideration. Both studies measured exposure and the outcome in a valid and reliable way. Suitable statistical procedure was reported in both studies. Confounding factors and strategies to adjust or control for them were unclear in Karakurum et al. (42) (Table 4).

**Table 4:** Risk of bias in cohort studies.

**References**	**Were the two groups similar and recruited from the same population?**	**Were the exposures measured similarly to assign people to both exposed and unexposed groups?**	**Was the exposure measured in a valid and reliable way?**	**Were confounding factors identified?**	**Were strategies to deal with confounding factors stated?**	**Were the groups/** **participants free of the outcome at the start of the study (or at the moment of exposure)?**	**Were the outcomes measured in a valid and reliable way?**	**Was the follow up time reported and sufficient to be long enough for outcomes to occur?**	**Were strategies to address incomplete follow up utilized?**	**Was appropriate statistical analysis used?**
Orta et al. (40)	Yes	Yes	Yes	Yes	Yes	Yes	Yes	Unclear	Unclear	Yes
Karakurum et al. (42)	Yes	Yes	Yes	Unclear	Unclear	Unclear	Yes	Unclear	Unclear	Yes

## Discussion

The aim of this review was to assess the link between anxiety and migraine, in order to determine whether (a) there is a usual comorbidity between migraine and anxiety and (b) if the incidence of anxiety is higher among migraineurs compared to non-migraineurs.

The results of the systematic review showed strong and consistent positive relationship between migraine and anxiety. The data analysis of included studies showed an average random effect of an average of RR of 1.63 (1.37–1.93) for two cohort studies and an average OR of 2.33 (2.20–2.47) for prevalence and cross-sectional studies of anxiety comorbidity among migraineurs compared to non-migraineurs or healthy participants. Clearly migraine and anxiety are comorbid, and the incidence of occurrence is almost four times higher compared to non-migraineurs. Our results are consistent with previous studies (47–49).

Furthermore, (41) reported that more than a third of their participants who were diagnosed with migraine reported positive reduction in their migraine attacks as a result of receiving pharmacological treatment for their anxiety. This suggests a joint predisposition or some related biological underpinnings in both migraine and anxiety (41). These results are also in line with the findings of our systematic review demonstrating the link between anxiety and migraine.

The current systematic review have important implications for future clinical practice. Firstly, the results highlight the need for concurrent assessment of migraineurs for both neurological symptoms of migraine and psychiatric symptoms associated with potential anxiety and depression. Secondly, in order to understand the etiology better, future studies should seek more information regarding the apparent onset of biological symptoms associating migraine with physiological measures of anxiety. Currently there is little biological information regarding the onset of clinical anxiety with regard to the onset of the migrainous events or vice versa. Experimental studies on the chronological association of migraine and anxiety would be expected to lead to clinical trials regarding the effectiveness of known treatments for both migraine severity and debilitating anxiety. Indeed, this will increase the likelihood of earlier detection and development of preventative strategies among people with migraine.

Lastly, when comorbidity is detected for migraine patients, treatment options should be considered that lead to improvements in both conditions (50, 51). Moreover, exploring the comorbidity of migraine with anxiety from a neurological perspective is likely to lead to greater understanding of the early etiology and aid in development of more effective treatment options. As acknowledged by the researchers (51), the comorbid symptoms appear to be an outcome of sensorimotor, interoceptive and cognitive adaptations. As a result, the migraine and anxiety comorbidity can be observed within the contexts of neurological and psychopharmacological settings (51). Further studies are needed on these treatment options. The high comorbidity of migraine and anxiety highlights the need for more research on the neurobiological causes of migraine and how best to manage its risk factors in a more effective way. Furthermore, there is a need to continue research into the psychiatric comorbidities of migraine to ascertain if there is a greater prevalence of comorbidity for anxiety in migraineurs with aura.

### Limitations

A limitation of the current systematic review is the small number of studies included in the review and not meeting the minimum required number of the studies for running a combined metanalysis. Furthermore, the nature of the observational studies included in this systematic review s limit the generalisability of the results. Finally, the diversity of the tools used to measure anxiety introduced a confounding factor to the statistical analysis.

## Conclusion

In the reported systematic review two critical results were found: (a) the comorbidity of migraine and anxiety is strong and significant and (b) the comorbidity of anxiety with migraine is significantly higher among migraineurs vs. non-migraineurs. This study also highlighted a need for concurrent screening or assessing migraine patients with anxiety tools. Biological assessments of anxiety among migraineurs is missing in the clinical settings.

## Data Availability Statement

The original contributions presented in the study are included in the article/supplementary materials, further inquiries can be directed to the corresponding author/s.

## Author Contributions

TW, SC, LK, and HK: substantial contributions to conception and design. LK, DE, and HK: acquisition of data or analysis and interpretation of data. LK, SC, TW, DE, HK, and AE: drafting the article or revising it critically for important intellectual content. All authors have agreed on the final version of the paper, contributed to the study, and development of the paper.

## Conflict of Interest

The authors declare that the research was conducted in the absence of any commercial or financial relationships that could be construed as a potential conflict of interest.

## References

[1] Stovner L Hagen K Jensen R Katsarava Z Lipton R Scher A. The global burden of headache: a documentation of headache prevalence and disability worldwide. Cephalalgia. (2007) 27:193–210. 10.1111/j.1468-2982.2007.01288.x 17381554

[2] Bakshi N Ross D Krishnamurti L. Presence of pain on three or more days of the week is associated with worse patient reported outcomes in adults with sickle cell disease. J Pain Res. (2018) 11:313–8. 10.2147/JPR.S150065 29445298 PMC5810514

[3] Burch RC Loder S Loder E Smitherman TA. The prevalence and burden of migraine and severe headache in the United States: updated statistics from government health surveillance studies. Headache. (2015) 55:21–34. 10.1111/head.12482 25600719

[4] Stovner LJ Andree C. Prevalence of headache in Europe: a review for the Eurolight project. J Headache Pain. (2010) 11:289–99. 10.1007/s10194-010-0217-0 20473702 PMC2917556

[5] Natoli J Manack A Dean B Butler Q Turkel C Stovner L. Global prevalence of chronic migraine: a systematic review. Cephalalgia. (2009) 30:599-609. 10.1111/j.1468-2982.2009.01941.x 19614702

[6] Smitherman TA Penzien DB Maizels M. Anxiety disorders and migraine intractability and progression. Curr Pain Headache Rep. (2008) 12:224–9. 10.1007/s11916-008-0039-9 18796274

[7] Kessler RC Berglund P Demler O Jin R Merikangas KR Walters EE. Lifetime prevalence and age-of-onset distributions of dsm-iv disorders in the national comorbidity survey replication. Arch Genl Psychiatry. (2005) 62:593–602. 10.1001/archpsyc.62.6.593 15939837

[8] Bouras NHG Psychiatric and Behavioral Disorders in Intellectual and Developmental Disabilities. 2nd ed St Ives: Cambridge University Press (2007).

[9] Association AP Diagnostic and Statistical Manual of Mental Disorders. 5th ed Arlington, VA: American Psychiatric Publishing (2013).

[10] Fuller-Thomson E Jayanthikumar J Agbeyaka SK. Untangling the association between migraine, pain, and anxiety: examining migraine and generalized anxiety disorders in a canadian population based study. Headache. (2016) 57:375–90. 10.1111/head.13010 27991658

[11] Merikangas KR Stevens DE. Comorbidity of migraine and psychiatric disorders. Neurol Clin. (1997) 15:115–23. 10.1016/S0733-8619(05)70298-X 9058400

[12] McWilliams LA Goodwin RD Cox BJ. Depression and anxiety associated with three pain conditions: results from a nationally representative sample. Pain. (2004) 111:77–83. 10.1016/j.pain.2004.06.002 15327811

[13] Breslau N Davis GC Andreski P. Migraine, psychiatric disorders, and suicide attempts: an epidemiologic study of young adults. Psychiatry Res. (1991) 37:11–23. 10.1016/0165-1781(91)90102-U 1862159

[14] Breslau N Schultz L Lipton R Peterson E Welch KM. Migraine headaches and suicide attempt. Headache. (2012) 52:723–31. 10.1111/j.1526-4610.2012.02117.x 22404176

[15] Hamelsky SW Lipton RB. Psychiatric comorbidity of migraine. Headache. (2006) 46:1327–33. 10.1111/j.1526-4610.2006.00576.x 17040330

[16] Swartz KL Pratt LA Armenian HK Lee LC Eaton WW. Mental disorders and the incidence of migraine headaches in a community sample: results from the baltimore epidemiologic catchment area follow-up study. Arch Gen Psychiatry. (2000) 57:945–50. 10.1001/archpsyc.57.10.945 11015812

[17] Karimi L Hoppe D Burdick C Buultjens M Wijeratne T Crewther SG. Recent evidence regarding the association between migraine and suicidal behaviors: a systematic review. Front Neurol. (2020) 11:490. 10.3389/fneur.2020.00490 32655476 PMC7324711

[18] Sareen J. Anxiety disorders and risk for suicide: why such controversy? Depression Anxiety. (2011) 28:941–5. 10.1002/da.20906 22076969

[19] Dresler T Caratozzolo S Guldolf K Huhn JI Loiacono C Niiberg-Pikksööt T. Understanding the nature of psychiatric comorbidity in migraine: a systematic review focused on interactions and treatment implications. J Headache Pain. (2019) 20:51. 10.1186/s10194-019-0988-x 31072313 PMC6734261

[20] Lahmann C Henningsen P Brandt T Strupp M Jahn K Dieterich M. Psychiatric comorbidity and psychosocial impairment among patients with vertigo and dizziness. J Neurol Nurosurg Psychiatry. (2015) 86:302–8. 10.1136/jnnp-2014-307601 24963122

[21] Brandt T Grill E Strupp M Huppert D. Susceptibility to fear of heights in bilateral vestibulopathy and other disorders of vertigo and balance. Front Neurol. (2018) 9:406. 10.3389/fneur.2018.00406 29928252 PMC5997824

[22] Charles A Brennan KC. The neurobiology of migraine. Handb Clin Neurol. (2010) 97:99–108. 10.1016/S0072-9752(10)97007-3 20816413 PMC5494713

[23] Alvares GA Quintana DS Hickie IB Guastella AJ. Autonomic nervous system dysfunction in psychiatric disorders and the impact of psychotropic medications: a systematic review and meta-analysis. J Psychiatry Neurosci. (2016) 41:89–104. 10.1503/jpn.140217 26447819 PMC4764485

[24] Bajkó Z Szekeres C-C Kovács KR Csapó K Molnár S Soltész P. Anxiety, depression and autonomic nervous system dysfunction in hypertension. J Neurol Sci. (2012) 317:112–6. 10.1016/j.jns.2012.02.014 22425019

[25] Goadsby PJ Hoskin KL. Inhibition of trigeminal neurons by intravenous administration of the serotonin (5HT) 1B/D receptor agonist zolmitriptan (311C90): are brain stem sites therapeutic target in migraine? Pain. (1996) 67:355–9. 10.1016/0304-3959(96)03118-1 8951929

[26] Robinson OJ Vytal K Cornwell BR Grillon C. The impact of anxiety upon cognition: perspectives from human threat of shock studies. Front Hum Neurosci. (2013) 7:203. 10.3389/fnhum.2013.00203 23730279 PMC3656338

[27] Strickland M Yacoubi-Loueslati B Bouhaouala-Zahar B Pender SL Larbi A. Relationships between ion channels, mitochondrial functions and inflammation in human aging. Front Physiol. (2019) 10:158. 10.3389/fphys.2019.00158 30881309 PMC6405477

[28] Herman JP McKlveen JM Ghosal S Kopp B Wulsin A Makinson R. Regulation of the hypothalamic-pituitary-adrenocortical stress response. Comprehensive Physiol. (2011) 6:603–21. 10.1002/cphy.c150015 27065163 PMC4867107

[29] Peres MFP Mercante JP Tobo PR Kamei H Bigal ME. Anxiety and depression symptoms and migraine: a symptom-based approach research. J Headache Pain. (2017) 18:37. 10.1186/s10194-017-0742-1 28324317 PMC5360747

[30] Paliwal VK. Anxiety depression, and its relationship with migraine. J Neurosci Rural Pract. (2019) 10:4–5. 10.4103/jnrp.jnrp_321_18 30765961 PMC6337992

[31] Song T-J Cho S-J Kim W-J Yang KI Yun C-H Chu K. Anxiety and depression in tension-type headache: a population-based study. PLoS ONE. (2016) 11:e0165316. 10.1371/journal.pone.0165316 27783660 PMC5082613

[32] Fernandez-de-Las-Penas C Ambite-Quesada S Palacios-Cena M Guillem-Mesado A Guerrero-Peral A Pareja JA Catechol-O-methyltransferase (COMT) rs4680 Val158Met Polymorphism is associated with widespread pressure pain sensitivity and depression in women with chronic, but not episodic, tension-type headache. Clin J Pain. (2019) 35:345–52. 10.1097/AJP.0000000000000684 30614828

[33] Clark LA Watson D. Tripartite model of anxiety and depression: psychometric evidence and taxonomic implications. J Abnormal Psychol. (1991) 100:316–36. 10.1037/0021-843X.100.3.316 1918611

[34] Watson D O'Hara MW Simms LJ Kotov R Chmielewski M McDade-Montez EA Development and validation of the Inventory of Depression and Anxiety Symptoms (IDAS). Psychol Assess. (2007) 19:253–68. 10.1037/1040-3590.19.3.253 17845118

[35] Moola S Munn Z Sears K Sfetcu R Currie M Lisy K. Conducting systematic reviews of association (etiology): the Joanna briggs institute's approach. Int J Evid Based Healthc. (2015) 13:163–9. 10.1097/XEB.0000000000000064 26262566

[36] Munn Z Moola S Lisy K Riitano D Tufanaru C. Methodological guidance for systematic reviews of observational epidemiological studies reporting prevalence and cumulative incidence data. Int J Evid Based Healthc. (2015) 13:147–53. 10.1097/XEB.0000000000000054 26317388

[37] Suurmond R van Rhee H Hak T. Introduction, comparison, and validation of meta-essentials: a free and simple tool for meta-analysis. Res Synthesis Methods. (2017) 8:537–53. 10.1002/jrsm.1260 28801932 PMC5725669

[38] Higgins JPT G.S.e. Cochrane Handbook for Systematic Reviews of Interventions Version 5.1.0 [updated March 2011]. The Cochrane Collaboration (2011)

[39] Mercante JPP Peres MFP Bernik MA. Primary headaches in patients with generalized anxiety disorder. J Headache Pain. (2011) 12:331–8. 10.1007/s10194-010-0290-4 21298316 PMC3094648

[40] Orta OR Gelaye B Qiu C Stoner L Williams MA. Depression, anxiety and stress among pregnant migraineurs in a pacific-northwest cohort. J Affect Disord. (2015) 172:390–6. 10.1016/j.jad.2014.10.032 25451442 PMC4406774

[41] Senaratne R Van Ameringen M Mancini C Patterson B Bennett M. The prevalence of migraine headaches in an anxiety disorders clinic sample. CNS Neurosci Therapeutics. (2010) 16:76–82. 10.1111/j.1755-5949.2009.00103.x 20415837 PMC6493819

[42] Karakurum B Soylu O Karatas M Giray S Tan M Arlier Z. Personality, depression, and anxiety as risk factors for chronic migraine. Int J Neurosci. (2004) 114:1391–9. 10.1080/00207450490476002 15636352

[43] Lampl C Thomas H Tassorelli C Katsarava Z Lainez JM Lanteri-Minet M. Headache, depression and anxiety: associations in the Eurolight project. J Headache Pain. (2016) 17:59. 10.1186/s10194-016-0649-2 27245683 PMC4887397

[44] Oh K Cho S-J Chung YK Kim J-M Chu MK. Combination of anxiety and depression is associated with an increased headache frequency in migraineurs: a population-based study. BMC Neurol. (2014) 14:238. 10.1186/s12883-014-0238-4 25494868 PMC4279894

[45] Victor TW Hu X Campbell J White RE Buse DC Lipton RB. Association between migraine, anxiety and depression. Cephalalgia. (2010) 30:567–75. 10.1111/j.1468-2982.2009.01944.x 19614684

[46] Lampl C Thomas H Stovner LJ Tassorelli C Katsarava Z Lainez JM. Interictal burden attributable to episodic headache: findings from the Eurolight project. J Headache Pain. (2016) 17:9. 10.1186/s10194-016-0599-8 26879832 PMC4754227

[47] Tan HJ Suganthi C Dhachayani S Rizal AMM Raymond RA. The coexistence of anxiety and depressive personality traits in migraine. Singapore Med J. (2007) 48:307–10. 17384877

[48] Saunders EFH Nazir R Kamali M Ryan KA Evans S Langenecker S. Gender differences, clinical correlates, and longitudinal outcome of bipolar disorder with comorbid migraine. J Clin Psychiatry. (2014) 75:512–9. 10.4088/JCP.13m08623 24816075 PMC4211932

[49] Lee ST Park JH Kim M. Efficacy of the 5-HT1A agonist, buspirone hydrochloride, in migraineurs with anxiety: a randomized, prospective, parallel group, double-blind, placebo-controlled study. Headache. (2005) 45:1004–11. 10.1111/j.1526-4610.2005.05181.x 16109114

[50] Low NCP Merikangas KR. The comorbidity of migraine. CNS Spectrums. (2003) 8:433–44. 10.1017/S1092852900018745 12858133

[51] Balaban CD Jacob RG Furman JM. Neurologic bases for comorbidity of balance disorders, anxiety disorders and migraine: neurotherapeutic implications. Expert Rev Neurotherapeutics. (2011) 11:379–94. 10.1586/ern.11.19 21375443 PMC3107725

[52] Valentine JC Pigott TD Rothstein HR How many studies do you need? A primer on statistical power for meta-analysis. J Educ Behav Stat. (2010) 35:215–47. 10.3102/1076998609346961

